# Supplementary material for: Rapid turnover of life-cycle-related genes in the brown algae
Source: Genome Biol. 2019 Feb 14;20:35. doi: 10.1186/s13059-019-1630-6 (PMC6374913; doi:10.1186/s13059-019-1630-6)
Supplement: Supplementary file 3 — Protein alignments, corresponding Gblocks html file and CDS sequences for the lineage conserved gametophyte-biased genes. (ZIP 2109 kb) [file 13059_2019_1630_MOESM3_ESM.zip › Con_GA_alignments/Ec-10_000430.aa_ali.fasta-gb.htm]

Ec-10\_000430.aa.fasta.trans.aa\_ali.fasta


## Gblocks 0.91b Results

Processed file: **Ec-10\_000430.aa.fasta.trans.aa\_ali.fasta**  
Number of sequences: **2**  
Alignment assumed to be: **Protein**  
New number of positions: **558** (selected positions are underlined in blue)

```
                         10        20        30        40        50        60
                 =========+=========+=========+=========+=========+=========+
Ec-10_000430     MALGHAEGEGQEEKNIYSSSSSSSDEDGSGSDSSDDDDDSSSSGSSSSRSSSSGSGSDDD
MSTRG.18092.1    ------------------------------------------------------------
                                                                             


                         70        80        90       100       110       120
                 =========+=========+=========+=========+=========+=========+
Ec-10_000430     DDREEGSEDENFEVGFWDSLKAVLPWTEAGAKADYAEERLDAHHEFQEELDEALRIRKMV
MSTRG.18092.1    ------------------------------------------------------------
                                                                             


                        130       140       150       160       170       180
                 =========+=========+=========+=========+=========+=========+
Ec-10_000430     ETGLHDMALDRLMASTEGQMYHHFHKHRYQLEQTGEWTFVPPSVKRRWRHAVKKTDKVEK
MSTRG.18092.1    ------------------------------------------------------------
                                                                             


                        190       200       210       220       230       240
                 =========+=========+=========+=========+=========+=========+
Ec-10_000430     LRAYMAMRRDRFLRIRGLCRQVRNARLKSSDLLVTVEPESLIDENLLQVAGGGKKSKPAN
MSTRG.18092.1    ------------------------------------------------------------
                                                                             


                        250       260       270       280       290       300
                 =========+=========+=========+=========+=========+=========+
Ec-10_000430     PFGGARLKKCHWQNVNSKGEQCFCTNNRMVHPHRLFKDEFGVLIHDTLATCGWHATRCVS
MSTRG.18092.1    ------------------------------------------------------------
                                                                             


                        310       320       330       340       350       360
                 =========+=========+=========+=========+=========+=========+
Ec-10_000430     NSHGDDPVVISTANEGALCNECLVQLTKGPIRNTTAFGAPGVRFKNKRLGSADTDPGTGG
MSTRG.18092.1    ------------------------------------------------------------
                                                                             


                        370       380       390       400       410       420
                 =========+=========+=========+=========+=========+=========+
Ec-10_000430     EEFLLQKEKNGERAARAVQRTYRGYRARHLRAMVALEHPLRALLRVESAIVMQSLVRRFL
MSTRG.18092.1    ------------------------------------------------------------
                                                                             


                        430       440       450       460       470       480
                 =========+=========+=========+=========+=========+=========+
Ec-10_000430     ARTLVRQISAFRNMNATAIQKMARGMQARALFRRKRARKMVQRQMLFLLARAHVASAKVA
MSTRG.18092.1    ------------------------------------------------------------
                                                                             


                        490       500       510       520       530       540
                 =========+=========+=========+=========+=========+=========+
Ec-10_000430     RRIREEMRKKNAVALSFQRAWRGKKGREKAAMLRAITVRRHGAAITTQKFWRCYVARQEL
MSTRG.18092.1    ------------------------------------------------------------
                                                                             


                        550       560       570       580       590       600
                 =========+=========+=========+=========+=========+=========+
Ec-10_000430     GTRRVAHRREVWAAVAMQSTIRMFADRRRTQKMRREFDGAVRTLQPLVRGLLARVTARRE
MSTRG.18092.1    ------------------------------------------------------------
                                                                             


                        610       620       630       640       650       660
                 =========+=========+=========+=========+=========+=========+
Ec-10_000430     RALLGQVWGWLGTDTVTANGIYARFLPKNKYLVSRESAPLQQFSASQSASGNVDAGNYDE
MSTRG.18092.1    ------------------------------------------------------------
                                                                             


                        670       680       690       700       710       720
                 =========+=========+=========+=========+=========+=========+
Ec-10_000430     LQKGLGSTSSLVRSTLEALPGERHFTEKFDRDRTGTCSKVEFKIGLESFLREAGYPLQKE
MSTRG.18092.1    ------------------------------------------------------------
                                                                             


                        730       740       750       760       770       780
                 =========+=========+=========+=========+=========+=========+
Ec-10_000430     EAALIVKRYFDGDGFCAWHSFLNSYHNAGSKSSACARHGRLLCTLCIRTKQLGFKMCECG
MSTRG.18092.1    ------------------------------------------------------------
                                                                             


                        790       800       810       820       830       840
                 =========+=========+=========+=========+=========+=========+
Ec-10_000430     HVFDAHELVVGGSGPAKGETVRKQDQRGRVVPQKQLSALLSKGLSKTVVDRPINVEGCSA
MSTRG.18092.1    ------------------------------------------------------------
                                                                             


                        850       860       870       880       890       900
                 =========+=========+=========+=========+=========+=========+
Ec-10_000430     ASNLRRAQYTNNSPSQAALISAQRPNGFRVTGARGGTKTPPHATTSSRARRRWRRQKPGR
MSTRG.18092.1    ------------------------------------------------------------
                                                                             


                        910       920       930       940       950       960
                 =========+=========+=========+=========+=========+=========+
Ec-10_000430     GSRSITEEASQPGGITALSLAELSPPFFGRRTSADGADGSGAAALSPTATGDVAGFPKEN
MSTRG.18092.1    ------------------------------------------------------------
                                                                             


                        970       980       990      1000      1010      1020
                 =========+=========+=========+=========+=========+=========+
Ec-10_000430     LDMVRPANQGATSPNKRKITYIPSFQEARSQEKERRDASCAGRRGQAERAALAEMNSAVQ
MSTRG.18092.1    ------------------------------------------------------------
                                                                             


                       1030      1040      1050      1060      1070      1080
                 =========+=========+=========+=========+=========+=========+
Ec-10_000430     GKNAADYAAKLQDDIATNTSRVDGRLCLAPVMSKKELRHAVFLERHWCKLIKDIRNGATD
MSTRG.18092.1    ------------------------------------------------------------
                                                                             


                       1090      1100      1110      1120      1130      1140
                 =========+=========+=========+=========+=========+=========+
Ec-10_000430     PHLPISEAERMRVEGLMIPNKKRAQRLDDRLRALGFHARASGIYKTAASASATGTT---A
MSTRG.18092.1    -------------QGLMIPNKKRAQRLDDRLRVLGFHARASGIFNTGASASPVRETRGVV
                               ##########################################    


                       1150      1160      1170      1180      1190      1200
                 =========+=========+=========+=========+=========+=========+
Ec-10_000430     SGSATAKRRAPGQSSR---NGGGGGGERKDTSSSAGSRPHTQEEKQESMAKMVEACRKRR
MSTRG.18092.1    SPPSAAHGRGLKRSPSAGDNESGVRGEGRRGASAGGSTPLTQEEQQESMAKKVEASKRRR
                 ##############     #########################################


                       1210      1220      1230      1240      1250      1260
                 =========+=========+=========+=========+=========+=========+
Ec-10_000430     PGTAESEAGGTPEQSVRRKRRTVTGGEMESAVRLLKSRGESRARSPSTGAWQAVPTVEQA
MSTRG.18092.1    PGTEGTESDGIG-RSVRRKRRTVTGGEMESEARLVKLREGSRARSPSAGAVQAIPTPEQA
                 ##########    ##############################################


                       1270      1280      1290      1300      1310      1320
                 =========+=========+=========+=========+=========+=========+
Ec-10_000430     SKTAYGLLTAASANAAGVVQAPSGRGFRKHVCLHPGCGKVFHLKASADRHQEKEHRFRMR
MSTRG.18092.1    SKTAYELLTAASANAAGVIEAKSGRGVCRYVCMHPGCGKVFHLKASAADHQKKEHRFRKR
                 ############################################################


                       1330      1340      1350      1360      1370      1380
                 =========+=========+=========+=========+=========+=========+
Ec-10_000430     LAAPTPLTDQFMLPSWPSDGVPWSSG----LPAPGRGK-KHTSVSKGTVTACRGPAG---
MSTRG.18092.1    LAAPTPLTDQFMSASWPSDGVPWVPGSSSSRPLLPFGKSRGKNAPHGTLKATSSPKKRTG
                 ##########################                                  


                       1390      1400      1410      1420      1430      1440
                 =========+=========+=========+=========+=========+=========+
Ec-10_000430     ---RTPTK-TAKTVGAGG---SAGDTVLDRGDASLHARFACGVPGCSHRFPEARLLGLHL
MSTRG.18092.1    SAGGGKGEGSREKRGGGGRMSNSQAPSLGHDDSSLQARFTCGVPGCEHRFPEARLLGLHL
                                            #################################


                       1450      1460      1470      1480      1490      1500
                 =========+=========+=========+=========+=========+=========+
Ec-10_000430     RMGHNEFDLEKMKARRDGTVPTQTLDGVTVTAAIDVGGHVRASFLGAYRLVPPFQSPTGC
MSTRG.18092.1    RMGHSDFDLDRMKAAKEGTEPTSTLDGISATASIDVGGNLRASFLGTYRLVPPFQPPTGC
                 ############################################################


                       1510      1520      1530      1540      1550      1560
                 =========+=========+=========+=========+=========+=========+
Ec-10_000430     PDIPACGLHGRPKHGCQRCADIARQSAAVGADDGGEPEDGRPPMGWPLPPAKFYTRAMVV
MSTRG.18092.1    RDILTCDLHGRLKHGCRRCEEITQLAAAAGS--AGVPENDRAPLGLPLPPAKFYTRAMVV
                 ##############################    ##########################


                       1570      1580      1590      1600      1610      1620
                 =========+=========+=========+=========+=========+=========+
Ec-10_000430     VNHHGRPINLDLDCAEELRCPLLRFPLRSCNPSTGPSVEHRRADNATDDKAGGDALPDVD
MSTRG.18092.1    VVHNGRPVKLDLDCSQTLRCPLLRFPPSRAPSVECVSDTERRTPLSDASMEPEDHGRHRA
                 ##########################                                  


                       1630      1640      1650      1660      1670      1680
                 =========+=========+=========+=========+=========+=========+
Ec-10_000430     SVLPGFGDG-LQGGQEASRNLTASSGGSRKDLTDDGQG-ASAPKND-----TDGGSGSFP
MSTRG.18092.1    GYADDFDHISLRSRQADNHSAVDEALRSKIPADDDEQDQAESAAEDGVPGCSDGTAVGQP
                                            ##########               ########


                       1690      1700      1710      1720      1730      1740
                 =========+=========+=========+=========+=========+=========+
Ec-10_000430     ARSGPS------------IDACIDDNG----NDD---VLPQHFLTGGGIDPAGLGENNSH
MSTRG.18092.1    TRQNSCSTKIDQTATTAVVDVGIDAANQDDDEEDVRCVLSPRRRKFDENNERGSSDNERQ
                 ##                                                  ########


                       1750      1760      1770      1780      1790      1800
                 =========+=========+=========+=========+=========+=========+
Ec-10_000430     HQHEPQHHQN----THHQRHHQHQHQHHGALVPARLVAVLVDGSGKGWMAARRLWSVPDV
MSTRG.18092.1    QQRQQQEPQGQLSDQCHPRHLDRQCKQRGALVPARLVALLVDGSGKGWMAARRLWSVHAV
                 #########       ############################################


                       1810      1820      1830      1840      1850      1860
                 =========+=========+=========+=========+=========+=========+
Ec-10_000430     GHHALAEAGLTAGDVDREQEFFVATEVEYLPLSLVRDTCTVTVDRKKDVHSKQRSGVLPK
MSTRG.18092.1    GFPALTKAGLAMRDIDQDQEFFVGTEVEYLPLSLVWDTCTIKIDRRDDVHSKQRLGALPT
                 ############################################################


                       1870      1880      1890
                 =========+=========+=========+===
Ec-10_000430     SMR------------------------------
MSTRG.18092.1    PLRYVSHGVDSATGDILVDSEVKLREKRLDGGR
                 ###
```

```
Parameters used
Minimum Number Of Sequences For A Conserved Position: 2
Minimum Number Of Sequences For A Flanking Position: 2
Maximum Number Of Contiguous Nonconserved Positions: 8
Minimum Length Of A Block: 10
Allowed Gap Positions: None
Use Similarity Matrices: Yes
```

```
Flank positions of the 10 selected block(s)
Flanks: [1095  1136]  [1141  1154]  [1160  1210]  [1215  1346]  [1408  1530]  [1535  1586]  [1648  1657]  [1673  1682]  [1733  1749]  [1757  1863]  

New number of positions in Ec-10_000430.aa.fasta.trans.aa_ali.fasta-gb:  558  (29% of the original 1893 positions)
```
